# Effect of Carbon Content and Boronizing Parameters on Growth Kinetics of Boride Layers Obtained on Carbon Steels

**DOI:** 10.3390/ma15051858

**Published:** 2022-03-02

**Authors:** Andrijana Milinović, Vlatko Marušić, Pejo Konjatić, Nikolina Berić

**Affiliations:** Mechanical Engineering Faculty in Slavonski Brod, University of Slavonski Brod, 35000 Slavonski Brod, Croatia; vmarusic@unisb.hr (V.M.); pkonjatic@unisb.hr (P.K.); nberic@unisb.hr (N.B.)

**Keywords:** pack boronizing, growth kinetics, activation energy, modelling, carbon steels

## Abstract

Boronizing is a thermochemical treatment performed to produce hard and wear-resistant surface layers. In order to control the process and obtain boride layers with the desired properties, it is very important to know how the boronizing parameters and the chemical composition of the treated steel affect the boronizing. The aim of the present study is to investigate the influence of carbon content in carbon steels, boronizing temperature, and boronizing duration on the growth kinetics of boride layers. For this purpose, three carbon steels (C1y5, C45, and C70W2) were boronized in solid medium. The experimental results show that there is a linear relationship between the carbon content and the activation energy values, and between the carbon content and the frequency factors. In addition, a statistical analysis was performed to determine the contribution of each factor. The ANOVA showed that boronizing temperature has the highest effect on the boride layer thickness, followed by the boronizing duration, while the carbon content of the steel has the least effect on the boride layer thickness. Based on a regression model, an empirical equation was derived to estimate the thickness of the boride layer on carbon steels as a function of carbon content, boronizing temperature, and duration.

## 1. Introduction

Tools, machine parts, and many other technical components are frequently exposed to friction and wear, often at high temperatures and in corrosive environments. Under such operating conditions, surface properties are critical to their reliable and long service life. The increasing demand for wear and corrosion resistance promoted development and application of numerous surface treatments such as carburizing, nitriding, and boronizing. Among the mentioned treatments, boronizing stands out because the properties of the obtained layers are superior to those obtained by nitriding and carburizing, especially in terms of hardness (1600–2000 HV and 650–900 HV, respectively) [1].

Boronizing is a thermochemical surface treatment in which boron atoms diffuse from a boron-rich medium into the surface of the treated part. By interacting with the atoms of the base material, they form intermetallic compounds (borides) on the surface of the part. The resulting surface layer is extremely hard and improves abrasion wear resistance at room temperature and elevated temperatures. In addition, boride layers improve resistance to adhesion wear, the corrosion-erosion resistance in non-oxidizing dilute acids and alkaline media, oxidation resistance and resistance to the influence of liquid metals [2,3,4]. All this means that the service life of machine parts can be extended several times if the boronizing parameters and materials are properly selected. Çetin et al. investigated the effects of boronizing on the wear and corrosion behavior of pack-boronized AISI 904L superaustenitic steel. The boronized samples showed a significant increase (up to 40 times) in abrasive wear resistance compared to untreated samples [5]. Other studies in the field of tribology have also shown improved properties of boronized surfaces under conditions of dry sliding wear [6,7], tribo-corrosion [8], and erosion [9].

Boronizing can be applied to a variety of ferrous and non-ferrous materials [10,11]. Unlike other surface layers, which are flat and parallel to the surface, boride layers have a characteristic saw-tooth morphology, which may be more or less pronounced, depending on the base material. The more pronounced saw-tooth morphology is dominant in low-carbon or low-alloy steels. Boronizing of high-carbon or high-alloy steels results in the formation of less pronounced saw-tooth morphology or even flat boride layers. The morphology of the layer directly affects its adhesion to the surface. From a quality point of view, adhesiveness is one of the most important properties of the surface layers. During operation, poor adhesion can cause spalling of the layer, so taking this into account, pronounced saw-toothed layers are preferred [12,13,14,15,16,17,18,19]. Boronizing can be performed in solid, liquid, and gaseous media. Boronizing in solid media, also called pack-boronizing, is the most commonly used method [6,12,20,21]. Boronizing of steels is usually carried out at temperatures from 800 to 1000 °C and duration from 1 to 10 h [2,5,8,12,22]. Obtained surface layer may consist of one (Fe_2_B) or two borides (FeB/Fe_2_B). In case of dual-phased layer, the FeB phase forms on the outermost surface, while the Fe_2_B phase forms between FeB and the base material. FeB and Fe_2_B differ in their properties. The hardness of Fe_2_B ranges from 1400 to 1600 HV. The hardness of FeB is higher, ranging from 1800 to 2100 HV, but despite higher hardness values, FeB is undesirable because of its brittleness, so the formation of a layer consisting only of the Fe_2_B phase is preferred. In addition to the different hardness values, FeB and Fe_2_B borides also have different coefficients of thermal expansion, which can cause formation of microcracks at the FeB/Fe_2_B interface. The presence of these cracks and the high internal stresses can often lead to spalling of the FeB layer when high loads are applied or the component is subjected to a thermal shock [4,6,12,15]. The required thickness of the boride layer depends on the type of steel and the intended applications. In general, thin layers (15 to 20 μm) are used to protect against adhesive wear, while thick layers are recommended to protect against abrasive wear and as an erosion-corrosion protection. The optimum thickness is between 50 and 250 µm for low-carbon and low-alloy steels and between 25 and 76 μm for high-alloy steels [4,23,24].

The thickness of the boride layer depends on the process parameters (temperature, duration, boron potential of the medium), as well as on the chemical composition of the alloy [12]. Many studies have been conducted to determine how the boronizing parameters affect the growth of the boride layer, and consequently, several models of boride layer growth kinetics have been reported in the literature [13,20,25]. Most of these studies were based on the Arrhenius equation, which allows the determination of the frequency factor and the activation energy. Karakaş et al. investigated the growth kinetics of the boride layers obtained on AISI H13 and established empirical equations for the boride layer thickness [12]. Yang et al. studied boronizing kinetics on AISI H13 steel, where duplex boronizing treatment was conducted on annealed coarse-grained samples and annealed air blast shot peened samples. The activation energy determined for annealed air blast shot peened samples was lower than for coarse-grained samples [21]. Based on Fick’s second law, Delai et al. studied the growth kinetics of boride layers on 4Cr5MoSiV1 steel. They established a diffusion model for prediction of thicknesses of FeB and Fe_2_B layers in dependence on boronizing temperature and duration [25]. Kaouka et al. investigated boronizing kinetics as well as the microhardness and fracture toughness of boride layers obtained on SAE 1035 after boronizing in a slurry salt bath. Using Arrhenius equation, the activation energy for boronizing SAE 1035 steel was estimated [26]. Kayali investigated boronizing kinetics and determined the values of activation energy for boronizing of AISI P20 steel in microwave furnace and conventional atmospheric furnace [10]. The main objective of the study conducted by Kartal et al. was to investigate the growth kinetics of the layers formed during the electrochemical boronizing of low carbon AISI 1018 steel in molten salt electrolyte. As a result, the activation energy was determined to be 172.75 kJ/mol [27]. Ruiz-Trabolsi et al. investigated the influence of the amount of matter involved in the kinetics of the growth of boride layers on AISI 1018 steel. The results show that the layer thickness depends not only on the boronizing parameters but also on the sample size [20].

While many authors investigated boronizing kinetics using the Arrhenius equation, other authors based their research on other approaches. Campos et al. [28] investigated growth kinetics using dimensional analysis, Mebarek et al. [29] used a fuzzy neural network-based approach, while Velázquez-Altamirano et al. [30] took a stochastic approach and used the Markov chain to model growth kinetics. Statistical methods, in particular the response surface methodology (RSM), have also been used in the study of boronizing. The RSM methodology is used for investigation of the relationship between dependent and independent variables. This method allows mathematical modelling of the process, which enables optimization of the process, determination of optimal values for the factors, and prediction of the target response. The RSM has proven to be a powerful tool for the analysis of experiments [31,32,33]. Arguelles-Ojedaa et al. established the regression model for predicting the hardness of boride layers on ASTM F-75 alloy [33]. Yalamaç et al. derived the regression equation for prediction of the boride layer thickness on GGG 70 ductile cast iron [34]. Other authors also used statistical methods in their investigations of boride layer properties [35,36,37,38,39].

Most studies in the field of growth kinetics of boride layers are concerned with the dependence of the layer thickness on temperature and duration of boronizing. As far as the influence of the chemical composition of the base material is concerned, the studies are mostly limited to the examination of different types of steel (and other materials) boronized under the same parameters [1,14,18,19,28,40,41,42]. Thus, the layer thicknesses obtained with the same parameters are compared, which means that no functional correlation is found between the layer thickness and the chemical composition of the steel. In order to control the process and obtain layers with the desired properties, understanding the correlation between the boronizing parameters, the chemical composition of the substrate, and the boride layer thickness is crucial. The aim of this study is to comprehensively investigate the growth kinetics of boride layers on carbon steels and to develop a diffusion model that takes into account, not only the boronizing parameters, but also the chemical composition of treated steel. Expressions showing a functional relationship between the carbon content and the activation energy values and between the carbon content and the frequency factors were established. In addition, a statistical analysis was performed and the empirical model was developed to estimate the boride layer thickness as a function of the carbon content of the steel, the boronizing temperature, and the duration.

## 2. Materials and Methods

### 2.1. Experimental

In order to investigate the influence of chemical composition on boride layer growth kinetics, some of the results from previous studies were used in this study [43,44,45]. Investigation was conducted on three carbon steels C15, C45, and C70W2, whose chemical compositions are given in Table 1. It is known that the chemical composition of the substrate material influences boronizing results. Since carbon steels are the object of this study, the influence of chemical composition on boronizing kinetics was investigated through the influence of carbon content.

A number of boronizing parameters have an influence on the final properties of boride layer, and in this study, the influence of boronizing temperature and duration on the thickness of the boride layer was investigated. Before boronizing, dilatometric examinations were performed using the Netzsch 402 E dilatometer to determine α to γ and γ to α transformation temperatures. Boronizing temperatures of 870, 920, and 970 °C were selected to ensure diffusion in austenite for all three steels. Three different times were chosen for the duration (4, 6, and 8 h). According to selected parameters, 3^2^ factorial design of experiment with three repetitions of experiment was established and 27 specimens with dimensions ∅ 16 mm × 7 mm were prepared from each steel.

Prior to boronizing, the surfaces of the specimens were cleaned and sanded with emery paper up to 600 grit. Pack boronizing was carried out in a solid medium consisting of Hef-Durferrit Durborid 3 powder applicable for boronizing at temperatures between 850 and 1000 °C [46]. Specimens were placed in steel containers, boronized in an electric furnace (LHP laboratory chamber furnace, manufacturer ES, Samobor, Croatia) under atmospheric conditions, and cooled in air at the end of boronizing. After boronizing, all specimens were cross-sectioned, mounted in cold mounting acrylic resin and prepared for metallographic examination by grinding (with emery paper up to 1000 grit), polishing (using alumina), and etching (with 3% nital). The microstructures were characterized by optical microscopy using a Leica DM 2500 M microscope and a Leica Q550 MW imaging solution.

**Figure 1:**
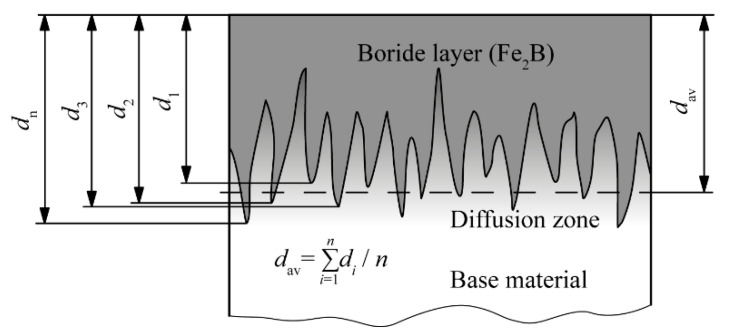
Determination of the boride layer thickness [43]. Reprinted with permission from ref. [43]. Copyright 2012 Mechanical Engineering Faculty in Slavonski Brod.

Boride layer thickness was determined as shown in Figure 1. Boride layer thickness was determined as the average value (*d*_av_) of the distances (*d*_1_, *d*_2_…*d*_n_) measured from the sample surface to the tips of the boride teeth.

### 2.2. Kinetic Study

The dependence between the thickness of the layer and the diffusion time is de-scribed by a parabolic law [1,10,13,18,20,21,43,44], expressed as follows:

(1) $$d^{2}=D\cdot t$$

where *d* is the thickness of diffusion layer (m), *D* is the growth rate constant (m^2^/s), and *t* is the diffusion duration (s). Since it is a quadratic equation, the solution is obtained by rooting it, where Equation (1) takes the form described below:

(2) $$d=\sqrt{D}\cdot \sqrt{t}$$

From Equation (2), it can be seen that the dependence between the layer thickness and the square root of the duration is linear. From the slope of the straight line, the square root of the growth rate constant can be calculated. The growth rate constant is temperature dependent, and this dependence is described by the Arrhenius equation [1,10,13,18,20,21,43,44]:

(3) $$D=D_{0\;}\cdot \;e^{-\frac{Q}{R\cdot T}}$$

where *D*_0_ is the frequency factor (m^2^/s), *Q* is the activation energy (kJ/mol), *T* is the diffusion temperature (K), and *R* is the universal gas constant (kJ/(mol·K)). The frequency factor represents the rate of molecular collisions in the reaction. Arrhenius Equation (3) shows that the frequency factor is the maximum possible rate constant when there is no energy barrier to overcome. Activation energy is the minimum amount of energy required for a chemical reaction to occur. When this amount of energy is provided, the reactant molecules can reach the transition state and overcome the energy barrier and consequently reaction could occur. If one takes the natural logarithm of the Arrhenius Equation (3), it takes a different form, which can be expressed as follows:

(4) $$\ln D=\ln D_{0}-\left(\frac{Q}{R}\right)\;\cdot \;\frac{1}{T}$$

According to Equation (4), the dependence between the natural logarithm of the growth rate constant and the reciprocal of the diffusion temperature can be expressed by a straight line whose slope is the quotient (*Q*/*R*) and ln*D*_0_ is the intersection of the straight line with the ordinate. Equation (5) is obtained by substituting Equation (3) into Equation (2).

(5) $$d=\sqrt{D_{0}\;\cdot t\;\cdot \;e^{-\frac{Q}{R\;\cdot T}}}$$

If the values of the frequency factor and the activation energy are known, Equation (5) can be used to calculate the thickness of the layer as a function of the temperature and the duration of diffusion.

### 2.3. Statistical Analysis

In order to determine which variable exerts the greatest influence on the thickness of the boride layer, a statistical analysis is performed as a part of this study. As already mentioned, each steel was boronized according to the 3^2^ full factorial design of experiment. One of the objectives of this study was to determine the functional relationship between the chemical composition of the steel and the boride layer thickness. Assuming that from the point of view of the chemical composition of a steel, carbon has the greatest influence on diffusion in non-alloy steels, the influence of chemical composition on boride layer thickness was analyzed through the influence of carbon content in the steel. With the introduction of carbon content as a factor, the 3^3^ factorial design of experiment was established, in which boride layer thickness is the dependent variable and carbon content, boronizing temperature, and duration are the independent variables.

## 3. Results

### 3.1. Microstructure

**Figure 2:**
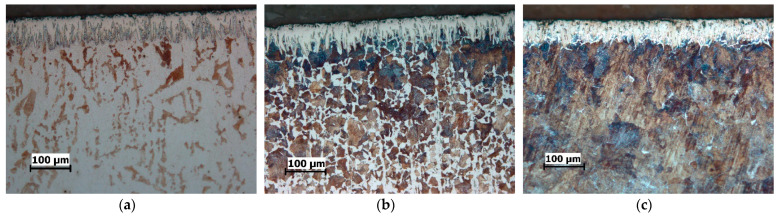
Microstructure of the boride layers obtained by boronizing at 870 °C for 4 h, magnification 200:1: (**a**) C15 steel; (**b**) C45 steel; (**c**) C70W2 steel.

**Figure 3:**
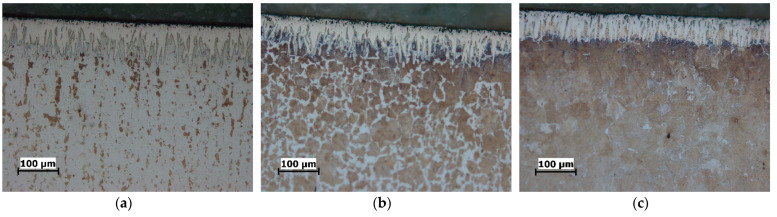
Microstructure of the boride layers obtained by boronizing at 870 °C for 8 h, magnification 200:1: (**a**) C15 steel; (**b**) C45 steel; (**c**) C70W2 steel.

**Figure 4:**
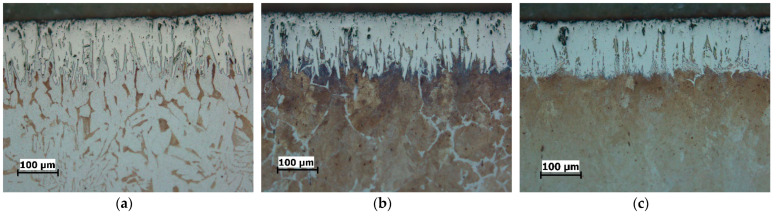
Microstructure of the boride layers obtained by boronizing at 970 °C for 4 h, magnification 200:1: (**a**) C15 steel; (**b**) C45 steel; (**c**) C70W2 steel.

The cross-sectional microstructure of the specimens boronized at different parameters is shown in Figure 2, Figure 3 and Figure 4. As can be seen, an increase in boronizing temperature and duration resulted in an increase in layer thickness. Moreover, for the same boronizing parameters, the boride layers on C15 steel are the thickest, followed by those on C45 and C70W2.

There is also a difference in the morphology of the layers. Although all the layers have a characteristic saw-toothed morphology, one can note the toothness is most pronounced on the C15 steel and least pronounced on the C70W2 steel.

In addition, porosity can be seen on the surface of the layers, which is more pronounced at higher temperatures. Darker sections within the diffusion zone, directly below the layer, can be observed in all three steels, and represent an increased carbide content compared to the core.

### 3.2. Boride Layer Growth Kinetics

#### 3.2.1. Results from Previous Studies

As mentioned above, the objective of the present study was to develop a diffusion model that takes into account not only the boronizing parameters but also the chemical composition of the treated steel. To achieve this, the results from earlier studies [43,44,45] were used as input data for a new, additional investigation conducted within this study. To facilitate the follow-up of the analysis and discussion, some of the results from previous studies are presented in this subsubsection.

Average boride layer thicknesses were determined in the manner described in Section 2. The boride layer thicknesses were calculated as the average of at least 50 measurements, and the results are presented in a Table 2 along with the standard deviations (SD).

For all three steels, it can be observed that increasing the boronizing temperature and/or duration leads to an increase in the layer thickness. Moreover, there is a difference between the thicknesses obtained on the different steels with the same boronizing parameters. The layers obtained on C15 steel are the thickest, followed by those on C45 steel, while those obtained on C70W2 steel are the thinnest. According to the values in Table 2, it is valid to assume that the thickness of the boride layer depends not only on the boronizing parameters, but also on the carbon content of these steels.

**Figure 5:**
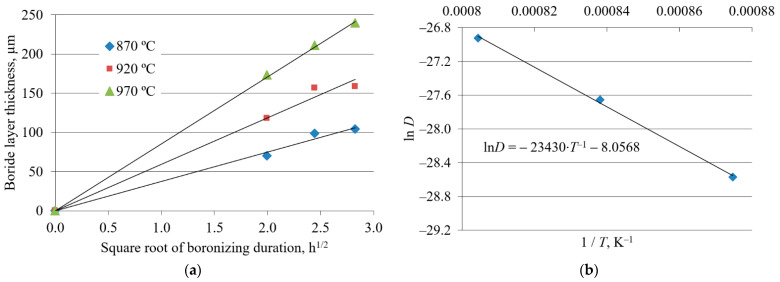
Growth kinetics of boride layers on C15 steel: (**a**) Boride layer thickness as a function of the square root of the boronizing duration; (**b**) natural logarithm of growth rate constant as a function of the reciprocal boronizing temperature [43]. Reprinted with permission from ref. [43]. Copyright 2012 Mechanical Engineering Faculty in Slavonski Brod.

**Figure 6:**
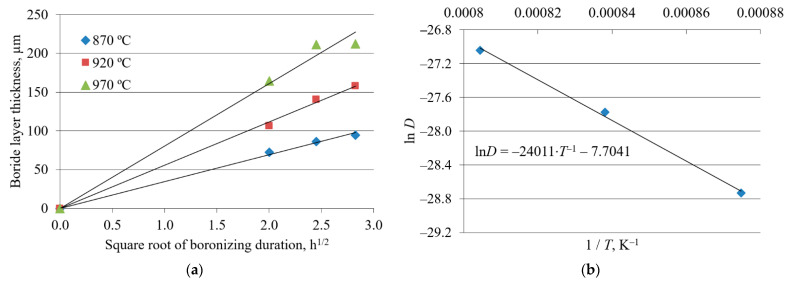
Growth kinetics of boride layers on C45 steel: (**a**) Boride layer thickness as a function of the square root of the boronizing duration; (**b**) natural logarithm of the growth rate constant as a function of the reciprocal boronizing temperature [44]. Reprinted with permission from ref. [44]. Copyright 2016 Croatian Metallurgical Society.

**Figure 7:**
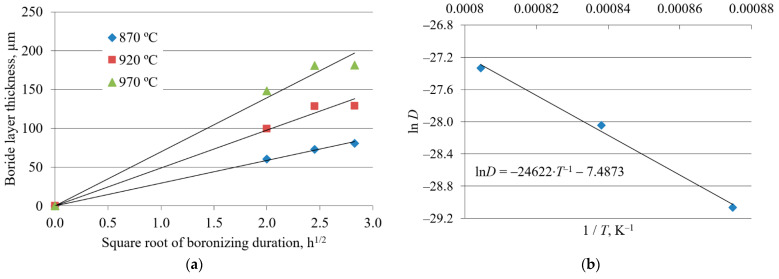
Growth kinetics of boride layers on C70W2 steel: (**a**) Boride layer thickness as a function of the square root of the boronizing duration; (**b**) natural logarithm of growth rate constant as a function of the reciprocal boronizing temperature [43]. Reprinted with permission from ref. [43]. Copyright 2012 Mechanical Engineering Faculty in Slavonski Brod.

The plots in Figure 5a, Figure 6a, and Figure 7a, constructed according to Equation (2), reveal a linear relationship between the boride layer thickness and the square root of boronizing duration for all three steels. The values of the growth rate constants were obtained from the slopes of the straight lines.

The plots of the natural logarithm of the growth rate constant versus the reciprocal diffusion temperature for all three steels show a linear relationship (Figure 5b, Figure 6b, and Figure 7b). For each steel, the value of the activation energy for boron diffusion was determined from the slope of the straight line, while the natural logarithm of the frequency factor was determined from the intersection of the extrapolated straight line with the ordinate. These values are given in Table 3.

#### 3.2.2. Dependence of the Frequency Factor and the Activation Energy on the Carbon Content

It is well-known that the chemical composition of the substrate material has an influence on the boride layer thickness. This is also evident from Equation (5), which shows that the thickness of the boride layer depends not only on the boronizing temperature and duration, but also on the frequency factor and activation energy. Table 3 shows that the C70W2 steel has the highest value of activation energy, followed by the C45 steel, while the C15 steel has the lowest value of activation energy.

The values of the frequency factor behave in the same way—the highest value is calculated for the C70W2 steel and the lowest for the C15 steel. As for the influence of chemical composition, in carbon steels, due to the low content of alloying elements, it can be assumed that the carbon content has the greatest influence on boron diffusion and, consequently, on the values of the frequency factor and activation energy. The C70W2 steel has the highest value of activation energy, followed by the C45 steel, while the C15 steel has the lowest value of activation energy. The values of the frequency factor behave in the same way—the highest value is calculated for the C70W2 steel and the lowest for the C15 steel.

**Figure 8:**
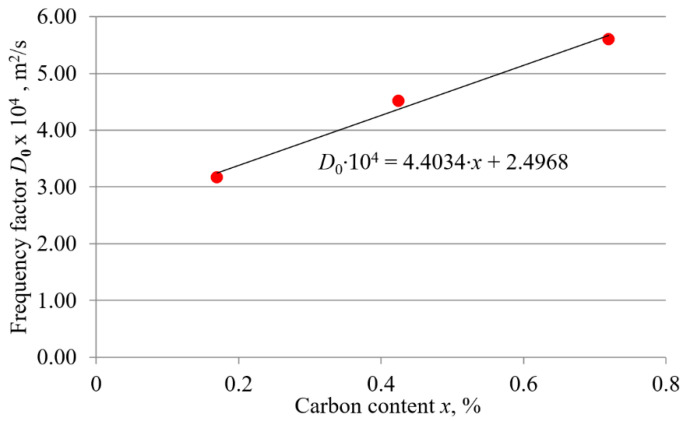
Relationship between the frequency factor and the carbon content of steel.

To determine if there is a functional relationship between the frequency factor and the carbon content, the values of the frequency factor given in Table 3 were plotted against the carbon content. The result is shown in Figure 8. Since the selected trend line agrees well with the data set, it can be concluded that for carbon steels, the value of the frequency factor can be estimated based on the carbon content. Figure 8 shows that the functional relationship between the frequency factor and carbon content of carbon steels tends to be linear, i.e., as the carbon content increases, the value of the frequency factor increases proportionally.

**Figure 9:**
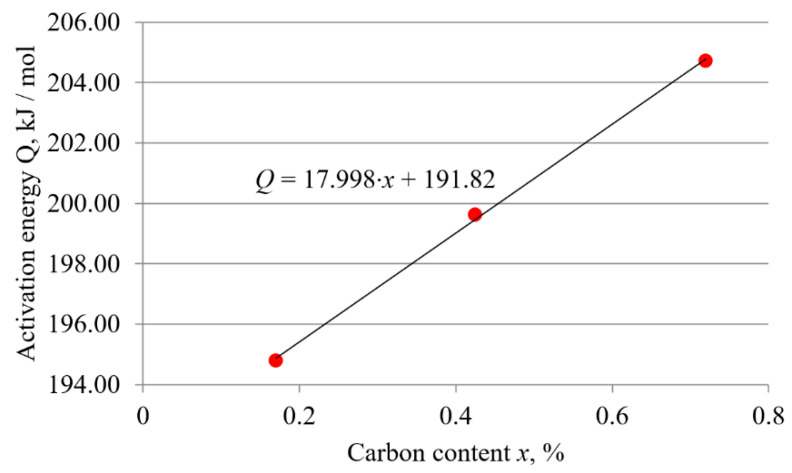
Relationship between the activation energy and the carbon content of steel.

To determine if there is a functional relationship between activation energy and the carbon content, the values of activation energy given in Table 3 were plotted against carbon content. The result is shown in Figure 9.

Since the selected trend line is a good approximation to the data set, it can be concluded that for carbon steels, the value of the activation energy can be estimated based on the carbon content. The diagram in Figure 9 shows that the relationship between the activation energy and the carbon content of carbon steels tends to be linear. As the carbon content increases, the value of the activation energy increases linearly. The following expressions were derived from the diagrams in Figure 8 and Figure 9:

(6) $$D_{0}=\left(4.4034\cdot x+2.4968\right)\cdot 10^{-4}$$

(7) Q=17.998 ·x+191.82

where *D*_0_ is the frequency factor (m^2^/s), *Q* is the activation energy (kJ/mol), and *x* is the carbon content of carbon steel (wt. %).

### 3.3. Statistical Analysis

Analysis of variance (ANOVA) and regression analysis were performed considering carbon content and boronizing temperature and duration as independent (or input) variables and boride layer thickness as dependent (or output) variable. Statistical analysis was performed by using a TIBCO Statistica software [47]. The main effect ANOVA was conducted to analyze the first-order effects of multiple categorical independent variables. Statistical significance was tested using the *F*-test and *p*-value. A high value of *F*-test means that the variance between groups is greater than the variance within groups, indicating that the difference in the thicknesses obtained is highly unlikely to be random and can be attributed to the effect of the independent variables. The level of statistical significance is expressed as a *p*-value, where the value *p* < 0.05 is statistically significant. It indicates that there is no relationship between the dependent variable and the independent variable with a probability of less than 5%, indicating that the independent variable influenced the dependent variable. A *p*-value greater than 0.05 is not statistically significant and indicates that there is no relationship between dependent and independent variable [48]. The ANOVA was conducted to test the significance between the means and the results are shown in Table 4. The *p*-values for all variables are less than 0.05, which makes them statistically significant. Regression analysis was performed to estimate the functional relationship between the variables. The second-order polynomial (quadratic model) was used for the regression model to approximate the nonlinear relationship. In order to avoid too many terms in the model, the insignificant terms were omitted.

Analysis of variance was performed for the reduced regression model, and the results are shown in Table 5. As can be seen, the *p*-value of all included terms is less than 0.05, so they are statistically significant.

**Figure 10:**
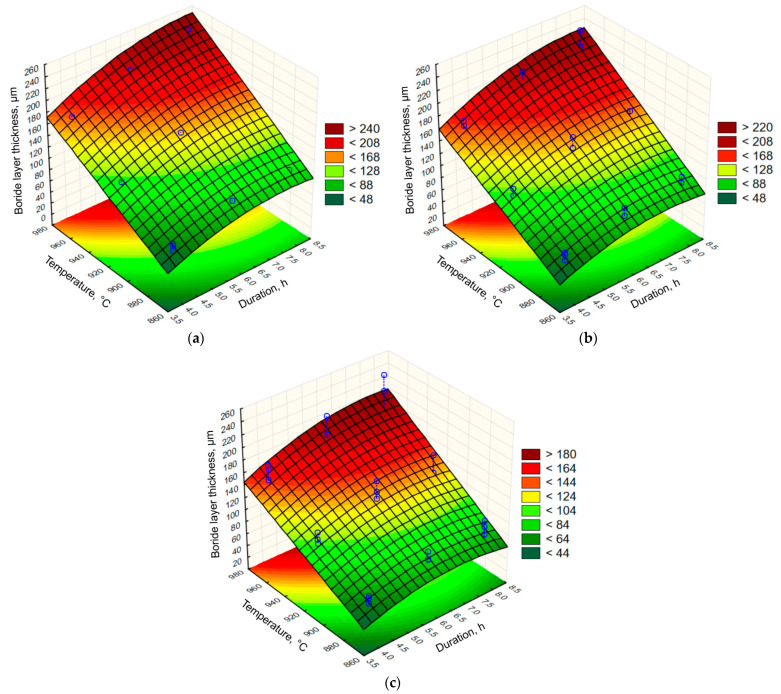
Relation between the boride layer thickness and the boronizing temperature and duration for: (**a**) C15 steel; (**b**) C45 steel; (**c**) C70W2 steel.

The regression coefficients were calculated for all terms included in the model, and the regression equation for the prediction of boride layer thickness was established as follows:

(8) $$d=-826.31+318.42\;\cdot x+0.88\;\cdot \;\vartheta -8.49\;\cdot \;t^{2}-0.34\;\cdot x\;\cdot \;\vartheta -8.84\;\cdot x\;\cdot t+0.06\;\cdot \;\vartheta \;\cdot t$$

where *d* is the average boride layer thickness (µm), *x* is the carbon content of the carbon steel (wt. %), *ϑ* is the boronizing temperature (°C), and *t* is the boronizing duration (h). The adjusted *R*^2^ is 0.98616, showing that the obtained model agrees with the observations. The graphical representations of the Equation (8) are given in Figure 10. The response surfaces in Figure 10a–c show the estimated boride layer thickness as a function of boronizing temperature and durations for C15, C45, and C70W2 steel, respectively.

## 4. Discussion

The optical examinations revealed that the microstructure consists of three zones: the boride layer, the diffusion zone, and the base material. The boride layer is the outermost, below the boride layer is the diffusion zone, and the base material is the zone unaffected by boronizing. The micrographs shown in Figure 2, Figure 3 and Figure 4 and the results given in Table 2 show that the thickness of the boride layers depends strongly on the boronizing temperature and duration, as well as on the steel grade. For all steels, higher boronizing temperatures and longer durations resulted in thicker layers. At a temperature of 970 °C, significantly thicker and denser boride layers were obtained than at 870 °C. It can also be observed that the temperature has a much greater effect on the thickness than the boronizing duration. This is in agreement with the results of other studies which have shown that the boronizing temperature has a greater effect on the thickness of the boride layer than the boronizing duration [3,6,10,12,23,29].

It is well-known that the chemical composition of the substrate material has an in-fluence on the properties of the boride layer. For carbon steels, due to the low content of alloying elements, it can be assumed that the carbon content has the main influence on boron diffusion. In view of this, the results in Table 2 show that the thickness of the layer is not only a function of the boronizing temperature and duration, but also a function of the carbon content. A higher carbon content in the steel resulted in a smaller thickness of the layer. A comparison of the microstructure of the layers obtained on the treated steels shows the difference in their morphology. The boride layers obtained on C15 steel have a pronounced saw-tooth morphology, while the layers obtained on C70W2 steel become denser and more compact, i.e., have minimal toothness. Namely, the increase in carbon content resulted in a less pronounced saw-tooth morphology. The main reason for this is that carbon is not soluble in the boride layer, so as the layer grows, carbon (and alloying elements in case of alloy steels) is forced inward from the surface. The increased carbon content in the diffusion zone acts as a barrier and impedes boron diffusion towards the substrate, resulting in the formation of thinner and smoother boride layers. The dependence of the layer thickness, as well as the morphology, on the chemical composition of the base material has been confirmed in studies by other authors. Atik et al. reported the formation of thicker layers on low-carbon steels compared to those on high-carbon steels, which can be attributed to the higher boron diffusion in low carbon steels [41]. Li et al. observed the formation of a compact and relatively smooth boride layer on Cr12Mn2V2 cast iron with high chromium content as a result of the high content of alloying elements [49]. Other authors have also confirmed in their studies that boronizing of steels with higher content of carbon and alloying elements leads to the formation of boride layers of less thickness and less toothness [2,3,6,12,13,23,29,42].

The darkly etched areas under the layer represent a higher share of carbides, which is also due to increased carbon content. The increased carbon content in the diffusion zone leads to the formation of an additional amount of carbides. The formation of an excess of carbides in the diffusion zone compared to the core has been confirmed by several researchers [2,3,16,21]. Porosity is observed at the surface of the layers, which is more pronounced at higher temperatures. Carrera-Espinoza et al. and Jain et al. reported porosity as a feature of boride layers obtained on low carbon steel [13,50]. Surface porosity has also been observed in studies by other authors [2,7,40].

Figure 5a, Figure 6a, and Figure 7a show the dependence of boride layer thickness on the square root of the boronizing duration. The linear relationship confirms that boronizing obeys the parabolic law (1) and that the kinetics of boride layer growth is controlled by boron diffusion. Yu et al. reported that the growth of boride layers at high temperatures follows a parabolic law, which was attributed to the diffusion of boron [51]. The diffusion nature of boronizing is also confirmed in other studies [1,4,20,27,42]. For each temperature, the C15 has the steepest slope, C45 has an intermediate slope, and C70W2 has the lowest slope of the straight lines. The slopes of the straight lines in Figure 5a, Figure 6a, and Figure 7a represent the square root of the growth rate constants at different temperatures. The decrease in the growth rate constant means a lower diffusion rate. As the carbon content increases, the growth rate constant decreases and the boride layer thickness tends to be smaller.

The values of activation energy and frequency factor for all three steels were obtained from the graphs in Figure 5b, Figure 6b, and Figure 7b. The activation energies reported by various researchers are listed in Table 6. As can be seen, the activation energy values in this study are comparable to those reported in the literature. However, there are some discrepancies between the results. These discrepancies could be due to the method used to measure the boride layer thickness, the surface preparation, the type of boronizing medium, the type of material used for containing the powder pack, possible spalling of the FeB layer, and the temperature ranges studied [12].

It can be seen from Table 3 that the value of the activation energy depends strongly on the carbon content of the carbon steel. As can be seen from Table 6, other studies have also shown that steels with higher carbon (and/or alloying elements) content have higher activation energy values. However, this study has shown that there is a functional relationship between these values. The plots in Figure 8 and Figure 9 show that, for carbon steels, both the activation energy and the frequency factor are affected by the carbon content. The linear relationship between them confirms the assumption that, the influence of chemical composition on boronizing kinetics dominates in carbon steels. As can be seen from the plots in Figure 8 and Figure 9, as the carbon content increases, both the frequency factor and the activation energy increase proportionally. Relationship between the activation energy and the carbon content of steel is shown in Figure 9. When the activation energy decreases, diffusion can flow more easily, resulting in a thicker layer. Compared to the other two steels, the C15 steel has the lowest activation energy, which confirms the enhancement of the boronizing kinetics. Accordingly, boron diffusion flows slightly slower in C45 steel and the slowest diffusion is achieved in C70 steel. Consequently, at the same boronizing temperatures and durations, the thickest layers are obtained on C15 steel and the thinnest on C70W2 steel.

Relationship between the frequency factor and the carbon content of steel is shown in Figure 8. The C15 steel has the lowest value of frequency factor, followed by the C45 steel, while the C70 W2 steel has the highest values of frequency factor. The higher the frequency factor is, the more frequent collisions will be, and the easier it is for reaction to occur. Figure 8 shows that C70W2 has the highest value of frequency factor among the steels tested. However, although high values of the frequency factor favor diffusion, the thickness of the layers obtained on this steel is the smallest. In contrast, the thickness of the layers obtained on the C15 steel is the highest, despite the lowest value of the frequency factor. The explanation for this comes from the Arrhenius Equation (3). As can be seen, an increase in the frequency factor proportionally increases the growth rate constant and thus the diffusion rate. On the other hand, an increase in the activation energy decreases the diffusion rate exponentially, so the effect of activation energy is quite large.

As a result of this study, Equation (6) and Equation (7) were established. Equation (6) is the corresponding equation for the plotted line in Figure 8. Equation (7) is the equation corresponding to the line shown in Figure 9. With the help of these expressions, it is possible to calculate the activation energy and frequency factor based on the carbon content of the hypoeutectoid carbon steels. Knowing these values and using Equation (5), it is possible to calculate the expected thickness of the boride layers with respect to the boronizing temperature and durations.

In this study, the experimental results were statistically analyzed using ANOVA and a regression analysis. The ANOVA was performed to determine whether the differences in boride layer thicknesses were due to chance or to the influence of independent variables, in this case, carbon content of steel, boronizing temperature, and duration. ANOVA showed that all three variables were highly statistically significant (*p* < 0.05). One way to measure the magnitude of the effect of the different variables in the analysis of variance is to use the partial eta squared, which describes the proportion of the variance attributable to a particular effect. The calculated values of the partial eta squared for carbon content, temperature, and duration are 0.763, 0.971, and 0.817, respectively. From these values, it can be concluded that the influence of boronizing temperature is the strongest, followed by duration and carbon content. The stronger influence of boronizing temperature compared to the influence of boronizing duration was also found in studies by other authors [3,10,12,23,29].

In order to obtain functional relationship between the variables, regression analysis was performed. Regression analysis is often performed to obtain empirical models describing certain material and engineering processes [20]. In general, regression models help to find the optimal combination of input variables to obtain the desired properties. Based on the experimental data, regression model described by Equation (8) was proposed to predict the boride layer thickness as a function of boronizing temperature, duration, and carbon content in a steel. This model is valid for boronizing of hypoeutectic carbon steels in the observed temperature and duration range. Based on ANOVA of the regression model (Table 5), the percentage contribution of each variable and its interaction on the boride layer thickness was determined. Boronizing temperature has the strongest effect on the response (81%), followed by duration (9.7%) and carbon content (5.1%). In comparison, the variable interactions as well as the square of the boronizing duration are statistically significant but have less influence on the boride layer thickness.

Figure 10 shows the three-dimensional plot of boride layer thickness as a function of boronizing temperature and duration for a fixed value of carbon content. It is obvious that the temperature has the strongest influence on the boride layer thickness. It can also be seen that the boride layer grows parabolically with time at a fixed temperature, which is consistent with Equation (1).

## 5. Conclusions

The aim of this study was to comprehensively investigate the growth kinetics of boride layers obtained on carbon steels. The investigation was carried out on three carbon steels (C15, C45, C70W2) pack boronized at temperatures of 870, 920, and 970 °C for 4, 6 and 8 h. Based on the test results, the following conclusions were drawn:

1. The boride layers obtained on all three steels have a characteristic saw-tooth morphology, but the degree of toothness is different. The boride layers on C15 steel exhibit the most pronounced saw-tooth morphology, while the layers on C70W2 exhibit minimal toothness.
2. For all three steels, increasing the boronizing temperature and duration resulted in an increase in boride layer thickness. It was also found that the thickness of the boride layer was strongly dependent on the carbon content of the carbon steel. For the same boronizing parameters, the boride layer thickness decreases as the carbon content of the steel increases. The thickest layers were produced on C15 steel and the thinnest on C70 steel.
3. The activation energy and frequency factor depend on the carbon content in the hypoeutectoid carbon steel. Both the activation energy and frequency factor increase linearly with increasing carbon content. The linear relationship confirms that in carbon steels, the influence of chemical composition has a dominant effect on boronizing kinetics. Based on the results, expressions showing the functional relationship between activation energy and carbon content and between frequency factor and carbon content were obtained. The obtained expressions allow the calculation of the activation energy and frequency factor based on the carbon content of hypoeutectoid carbon steel.
4. The analysis of variance showed that the influence of boronizing temperature, duration and carbon content of carbon steel on the boride layer thickness are highly statistically significant. The influence of boronizing temperature is strongest, followed by duration and carbon content.
5. Regression analysis was performed and through the analysis of variance of the regression model, it was found that the boronizing temperature has the strongest effect on the response, followed by the duration and carbon content. Compared to them, the variable interactions as well as the square of boronizing duration are statistically significant, but have less influence on the boride layer thickness.
6. The regression model based on the design of experiment was built to predict the boride layer thickness as a function of the carbon content of the hypoeutectic carbon steel and the boronizing temperature and duration. This model is valid for the boronizing of hypoeutectic carbon steels in the observed temperature and duration range.

## Figures and Tables

**Table 1:** Chemical composition of boronized steels, wt.% [43,44,45].

	C	Si	Mn	P	S	Cu
C15	0.17	0.24	0.38	0.023	0.005	0.04
C45	0.425	0.255	0.78	0.013	0.035	-
C70W2	0.72	0.211	0.286	0.014	0.017	-

**Table 2:** The average values of the boride layers obtained on C15, C45, and C70W2 steels, μm.

Boronizing Temperature, °C	Boronizing Duration, h	C15 [43,45]	SD	C45 [44]	SD	C70W2 [43]	SD
870	4	70	10.4	73	11.5	61	8.6
	6	98	16.4	86	15.9	73	8.8
	8	104	7.8	94	16.0	81	9.9
920	4	118	13.3	107	13.3	100	7.8
	6	157	12.7	140	12.3	129	9.1
	8	158	13.5	158	11.3	129	10.9
970	4	173	12.4	165	12.0	148	9.1
	6	211	19.3	211	15.5	181	11.5
	8	239	13.3	213	18.9	181	10.3

**Table 3:** Values of the frequency factor and the activation energy [43,44].

Steel	Frequency Factor *D*_0_, m^2^/s	Activation Energy *Q*, kJ/mol
C15	3.17 × 10^−4^	194.80
C45	4.51 × 10^−4^	199.63
C70W2	5.6 × 10^−4^	204.71

**Table 4:** Analysis of variance for the boride layer thickness.

	Sum of Squares SS	Degrees of Freedom df	Mean Square MS	*F*-Value	*p*-Value	Partial eta Squared
Intercept	322,940.1	1	322,940.1	3744.233	0,000,000	0.997
Carbon content	3334.7	1	3334.7	38.663	4.46 × 10^−5^	0.763
Temperature	34,816.4	2	17,408.2	201.834	5.79 × 10^−10^	0.971
Duration	4620.8	2	2310.4	26.787	3.76 × 10^−5^	0.817
Error	1035	12	86.3			

**Table 5:** Analysis of variance for the reduced regression model.

	Sum of Squares SS	Degrees of Freedom df	Mean Square MS	*F*-Value	*p*-Value
(1) Carbon content	3383.76	1	3383.76	96.556	6.97 × 10^−9^
(2) Temperature	53,302.59	1	53,302.59	1521.002	1.34 × 10^−19^
(3) Duration	6412.00	1	6412.00	182.968	3.34 × 10^−11^
Duration ^2^	740.74	1	740.74	21.137	1.97 × 10^−4^
(1) × (2)	266.78	1	266.78	7.613	1.25 × 10^−2^
(1) × (3)	284.07	1	284.07	8.106	1.03 × 10^−2^
(2) × (3)	432.00	1	432.00	12.327	2.34 × 10^−3^
Error	665.84	19	35.04		
Total Sum of Squares	65,844.74	26			

**Table 6:** Comparison of the activation energies.

Material	Boronizing Medium	Activation Energy, kJ/mol	References
AISI 440C	Solid media	340.4	[52]
AISI 316	Solid media	199.0	[53]
AISI 316	Plasma paste	250.8	[39]
AISI D2	Molten salt	170.0	[18]
AISI H13	Solid media	186.2	[54]
AISI H13	Solid media	284.2	[12]
AISI 52100	Solid media	269.6	[52]
AISI 5140	Molten salt	223.0	[18]
AISI 4340	Molten salt	234.0	[18]
AISI 4140	Molten salt	215.0	[55]
AISI P20	Solid media	200.0	[56]
AISI P20	Solid media	256.5	[10]
AISI W1	Solid media	171.2	[57]
AISI W110	Solid media	165.0	[1]
AISI 1045	Solid media	186.0	[1]
AISI 1040	Solid media	168.0	[56]
SAE 1035	Molten salt	227.51	[26]
SAE 1020	Solid media	183.15	[35]
AISI 1018	Solid media	148.3	[20]
AISI 1018	Molten salt	172,8	[27]
AISI 1018	Molten salt	172,8	[27]
AISI 1015	Solid media	190.0	[1]
Armco iron	Paste	157.0	[24]

## Data Availability

Not applicable.
